# Nutritional Modulation of Melatonin‐SIRT1 Signaling by Octanoic Acid‐Rich Enteral Nutrition Protects Against Radiation‐Induced Intestinal Injury

**DOI:** 10.1002/fsn3.71465

**Published:** 2026-01-27

**Authors:** Chenxi Zhou, Xiaohua Li, Chungen Xing, Chun Cao

**Affiliations:** ^1^ Department of General Surgery, the Second Affiliated Hospital of Soochow University Suzhou China; ^2^ Department of Thyroid and Breast Surgery, Suzhou Wuzhong People's Hospital Suzhou China; ^3^ State Key Laboratory of Radiation Medicine and Protection Soochow University Suzhou China

**Keywords:** enteral nutrition, melatonin, octanoic acid, radiation‐induced intestinal injury, SIRT1/PGC‐1α/PPARγ pathway

## Abstract

Radiation‐induced intestinal injury (RIII) is a common complication in patients under radiotherapy for abdominopelvic and retroperitoneal malignancies, significantly impairing quality of life and overall survival. However, the therapeutic effect of standard enteral nutrition (EN) is limited. This study aimed to investigate the protective role and potential mechanisms of octanoic acid (OA)‐rich EN in RIII. C3H/HeN mice were randomly assigned to four groups: Sham, Radiation (RI), RI + EN and RI + OA‐rich EN to investigate the impacts of OA‐rich EN. Then mice were randomly assigned to five groups: Sham, RI, RI + OA‐rich EN, RI + OA‐rich EN + Luzindole, RI + OA‐rich EN + EX527 to examine whether OA‐rich EN alleviated RIII through the melatonin‐silent information regulator 1 (SIRT1) pathway. We evaluated the intestinal histopathology, apoptosis, tight junction protein expression and permeability. Moreover, melatonin and inflammatory cytokine levels were measured in the intestine and serum. SIRT1/Peroxisome Proliferator‐Activated Receptor Gamma Coactivator 1‐Alpha (PGC‐1α)/Peroxisome Proliferator‐Activated Receptor Gamma (PPARγ) pathway was also assessed. OA‐rich EN promoted melatonin secretion in the intestine and serum, activated the SIRT1/PGC‐1α/PPARγ pathway, markedly improved intestinal histopathology, and significantly reduced levels of inflammatory factors in intestine and serum. These beneficial effects were greater than EN alone. Furthermore, these beneficial effects were abolished when OA‐rich EN was co‐administered with either a melatonin antagonist or a SIRT1 inhibitor. This is the first confirmation that OA‐rich EN alleviated RIII by promoting melatonin secretion, which in turn activated the SIRT1/PGC‐1α/PPARγ pathway. Our findings highlight OA‐rich EN as a promising nutritional strategy to improve intestinal health and reduce treatment‐related complications in patients receiving abdominal radiotherapy.

## Graphical abstract

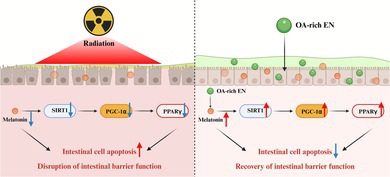

## 1 Introduction

Radiation‐induced intestinal injury (RIII) is a common intestinal complication in patients undergoing radiotherapy for abdominopelvic and retroperitoneal malignancies (Wu et al. 2025). This condition not only significantly impacts patients' quality of life but also, in severe cases, leads to the interruption of radiotherapy schedules, thereby compromising tumor control efficacy and even endangering patients' lives (Moraitis et al. 2025). Currently, there is a lack of specific therapeutic methods for RIII in clinical practice, leading to an enormous challenge in the fields of radiation oncology and clinical nutrition (Kong et al. 2023).

Among existing intervention strategies, nutritional support is recognized as an effective adjunctive therapy for RIII (Fan et al. 2025; Shao et al. 2014). Enteral nutrition (EN), as a core clinical nutritional intervention, plays a vital role in safeguarding gastrointestinal health, particularly in patients with intestinal injury (Martín Martín et al. 2025). By providing nutrients directly through the gastrointestinal tract, EN preserves the structural and functional integrity of intestinal epithelial cells, stimulates mucosal proliferation, and enhances the expression of tight junction proteins (Duan et al. 2025). For patients undergoing abdominal radiotherapy, the intestinal mucosa is severely damaged, leading to reduced nutrient absorption capacity and increased risk of malnutrition (Shao et al. 2014). EN not only supplies essential nutrients but also modulates intestinal metabolism and immune function, creating a favorable microenvironment for intestinal barrier repair (Fan et al. 2025; Shao et al. 2014).

Medium‐chain fatty acids (MCFAs) are a class of nutrients with unique nutritional characteristics that have garnered significant academic attention in recent years (Cao et al. 2025; Nagao and Yanagita 2010; Schönfeld and Wojtczak 2016). Unlike long‐chain fatty acids, MCFAs are rapidly absorbed in the small intestine without the need for bile acid emulsification or chylomicron formation, enabling quick transport to the liver for oxidation and energy production (Cao et al. 2025). This rapid metabolic feature makes MCFAs an efficient energy source, especially for individuals with impaired intestinal absorption function. Additionally, MCFAs exhibit inherent anti‐inflammatory properties by regulating immune cell responses and reducing the release of pro‐inflammatory cytokines, which is crucial for maintaining intestinal immune homeostasis (Nagao and Yanagita 2010; Schönfeld and Wojtczak 2016).

Melatonin is a neuroendocrine hormone primarily secreted by the pineal gland, involved in regulating circadian rhythms, sleep–wake cycles, and immune homeostasis (Vasey et al. 2021). In recent years, it has gained widespread attention as a dietary supplement for improving sleep disorders. Beyond sleep regulation, studies have also indicated that melatonin possesses significant antioxidant, anti‐inflammatory, and immunomodulatory activities (Minich et al. 2022; Reiter et al. 2016; Tan et al. 2015). Melatonin has also been shown to mitigate the impact of ionizing radiation on healthy animal tissues and alleviate the clinical manifestations induced by radiation, consequently extending survival times (Zetner et al. 2016). Silent information regulator 1 (SIRT1) is an NAD^+^‐dependent deacetylase that regulates various biological processes at the post‐transcriptional level (Cantó and Auwerx 2012), playing a crucial role particularly in inflammatory responses (Kim and Lee 2007). SIRT1 influences energy metabolism, stress responses, and cell survival by deacetylating histone and non‐histone substrates, such as Peroxisome Proliferator‐Activated Receptor Gamma Coactivator 1‐Alpha (PGC‐1α) (Tang et al. 2017; Li et al. 2014). PGC‐1α, a classical downstream target of SIRT1, is a core transcriptional coactivator regulating mitochondrial biogenesis and energy metabolism (de Prati et al. 2005). Activation of SIRT1 promotes autophagy and inhibits apoptosis, thereby protecting intestinal epithelial cells against radiation‐induced damage (Qin et al. 2021).

Among MCFAs, octanoic acid (OA) stands out due to its higher bioavailability and more potent regulatory effects on hormone secretion compared to other MCFAs (Zhang, Xu, et al. 2024; Zhang, Li, and Cao 2024; Lemarié et al. 2018). We previously reported that OA‐rich EN alleviated sepsis induced acute intestine injury through Peroxisome Proliferator‐Activated Receptor Gamma (PPARγ)/Signal Transducer and Activator of Transcription 1/Myeloid differentiation primary response 88 pathway and dextran sulfate sodium salt induced inflammatory bowel disease via the PPARγ/STAT‐1/STAT‐6 pathway mediated regulation of intestinal macrophage polarization (Tang et al. 2023; Xue and Cao 2025). Emerging evidence suggests that nutritional factors can modulate the synthesis and release of neuroendocrine hormones, and OA may act as a nutritional regulator to influence melatonin production (Zhang, Xu, et al. 2024; Zhang, Li, and Cao 2024). The potential metabolic link between OA and melatonin provides a critical nutritional mechanism for understanding how OA‐rich EN exerts protective effects against RIII, highlighting the importance of nutrient‐hormone interactions.

This study aims to determine whether OA‐rich EN confers superior protection against RIII, compared with EN alone. It further aims to decipher the underlying mechanism, with a particular focus on the role of melatonin and its downstream signaling pathway, while highlighting the nutritional significance of OA‐rich EN as a clinical intervention strategy.

## 2 Materials and Methods

### 2.1 Animals

Eight‐week‐old male C3H/HeN mice were used in this study. Mice were housed under specific pathogen‐free conditions and had free access to a standard diet and drinking water. All animal experiments were conducted in accordance with protocols approved by the Institutional Animal Care and Use Committee of Soochow University (Suzhou, China).

### 2.2 Experimental Design

Following anesthesia induction, mice were positioned at a source‐to‐skin distance of 100 cm for abdominal irradiation. The delivery of a single 10 Gy dose was accomplished using a 4‐MeV electron beam generated by an X‐RAD 320iX Biological Irradiator (Precision X‐ray, USA) at a dose rate of 2 Gy/min. A 3 cm × 2 cm abdominal area extending from the thigh base was targeted, while the remainder of the body was shielded with a 5‐cm‐thick lead block. To ensure dosimetric accuracy, the delivered radiation was verified with an external ion chamber dosimeter.

The EN solution (Abbott, Chicago, IL, USA) was provided at a dosage of 100 kcal/kg/day. For OA‐rich EN, the EN solution was supplemented with OA (Aladdin, Shanghai) to achieve an isocaloric diet, with the total 100 kcal/kg/day consisting of 95.5 kcal/kg/day from EN and 4.5 kcal/kg/day from OA. To ensure consistent energy intake across all cohorts, the Sham and RI groups were fed a standard diet isocaloric to the intervention groups.

**FIGURE 1:**
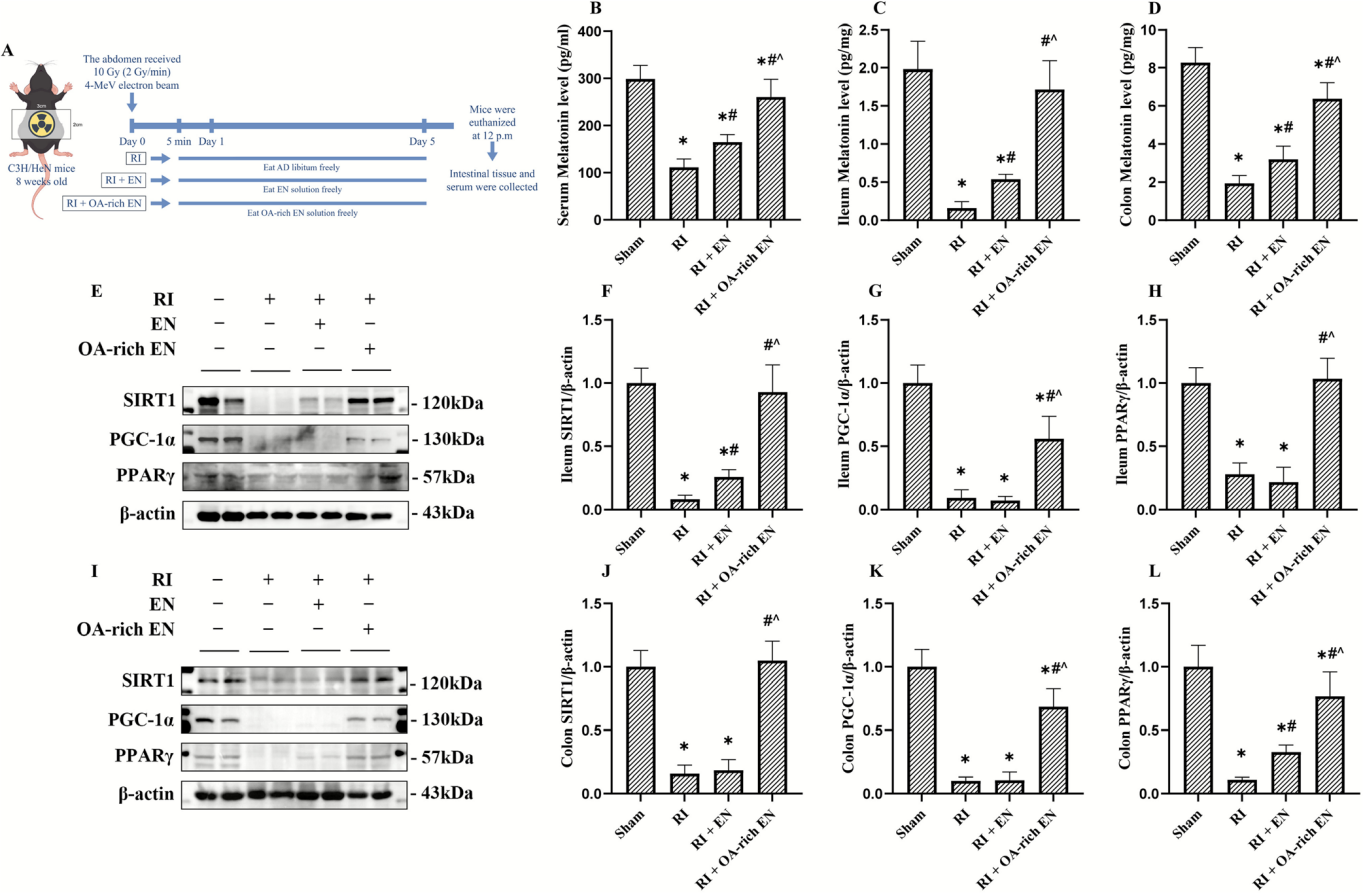
OA‐rich EN promoted melatonin secretion via nutrient‐hormone interaction and activated SIRT1/PGC‐1α/PPARγ pathway after RIII. The grouping and treatment protocols for the mice were schematically represented (A). The levels of melatonin were measured in the serum (B), ileum (C), and colon (D) by ELISA. The expression levels of SIRT1, PGC‐1α, and PPARγ in the ileum (E) and colon (I) were evaluated by western blotting. The relative protein expression of SIRT1, PGC‐1α, and PPARγ in the ileum (F–H) and colon (J–L) was normalized to that of β‐actin. Data are presented as the mean ± standard error of the mean. *p < 0.05 versus the Sham group. #*p* < 0.05 versus the RI group. ^*p* < 0.05 versus the RI + EN group. EN, enteral nutrition; OA, octanoic acid; PGC‐1α, Proliferator‐Activated Receptor Gamma Coactivator 1‐Alpha; PPARγ, Peroxisome Proliferator‐Activated Receptor Gamma; RI, radiation; SIRT1, Silent Information Regulator Transcript 1.

Male mice were randomly assigned to four groups (*n* = 8 per group): (1) Sham (No irradiation); (2) RI (Irradiation); (3) RI + EN (Receiving standard EN after RI); (4) RI + OA‐rich EN (Receiving standard EN supplemented with OA after RI). All irradiated groups underwent abdominal X‐ray (10 Gy) under identical conditions, with nutritional interventions administered for 5 days (Figure 1A).

To elucidate the underlying mechanisms, a new cohort of mice was utilized for validation. Mice were randomly assigned to five groups (*n* = 8 per group): (1) Sham; (2) RI; (3) RI + OA‐rich EN; (4) RI + OA‐rich EN + Luzindole (30 mg/kg/day, MCE, HY‐101254); (5) RI + OA‐rich EN + EX527 (10 mg/kg/day, MCE, HY‐15452). The respective inhibitors were administered intraperitoneally 5 min post‐irradiation, while other treatments remained consistent with the first experimental phase (Figure 5A). The dosages of Luzindole and EX527 are based on previous literature (Lee et al. 2025; Zhao et al. 2023).

### 2.3 Melatonin Measurement

Melatonin levels in the serum and intestine were measured with commercial ELISA kits (Elabscience, Wuhan, China) following the protocols.

### 2.4 Histopathological Evaluation

After fixation in 10% neutral buffered formalin overnight, ileum and colon underwent standard paraffin processing. The embedded tissues were sectioned at 5 μm for histological staining with both Hematoxylin–eosin (H&E) and Periodic Acid‐Schiff (PAS). All sections were subsequently imaged under a light microscope, and a blinded histopathological assessment of the damage was conducted by an experienced pathologist.

### 2.5 Inflammatory Cytokine Measurement

The levels of interleukin‐6 (IL‐6), IL‐1β, and tumor necrosis factor‐α (TNF‐α) in the serum and intestine were quantified using commercial ELISA kits (RayBiotech, Atlanta, GA, USA) according to the manufacturer's instructions.

### 2.6 Western Blot Analysis

Collected intestinal tissue samples were homogenized on ice and subsequently lysed in RIPA buffer to extract total protein. Proteins from each experimental group were separated by electrophoresis at 90 V for 2 h and then transferred onto a polyvinylidene difluoride membrane at 120 V for 90 min in a cold transfer buffer. The membrane was blocked with 5% non‐fat milk and then incubated with primary antibodies against PPARγ (Cell Signaling Technology, CST #2443, 1:1000), SIRT1 (CST #9475, 1:1000), PGC‐1α (CST #2178, 1:1000), B‐cell lymphoma‐extra large (Bcl‐xL; CST #2764, 1:1000), Bcl‐2‐associated X protein (BAX; CST #2772, 1:1000), Cleaved Caspase‐3 (CST #9661, 1:1000), Zonula Occludens‐1 (ZO‐1; Proteintech #21773, 1:1000), Occludin (CST #91131, 1:1000), and β‐actin (CST #4967, 1:5000) overnight at 4°C. Following three washes with TBST, incubation with HRP‐conjugated secondary antibodies was carried out for 1 h at room temperature. Target proteins were ultimately visualized via enhanced chemiluminescence detection.

### 2.7 Immunohistochemistry

To evaluate intestinal barrier function, immunohistochemical analysis of ZO‐1 and Occludin was performed on paraffin‐embedded intestinal sections. Following deparaffinization, rehydration, and antigen retrieval, endogenous peroxidase activity was blocked with 3% H_2_O_2_. The sections were then incubated overnight at 4°C with primary antibodies against ZO‐1 (Proteintech #21773, 1:100) or Occludin (CST #91131, 1:100). Then, sections were incubated with secondary antibodies at room temperature for 30 min. Protein localization was finally visualized and documented using light microscopy.

### 2.8 Fluorescein Isothiocyanate‐Labeled Dextran

Intestinal barrier function was quantitatively evaluated by measuring the systemic translocation of a macromolecular tracer, fluorescein isothiocyanate‐labeled dextran (molecular weight: 4‐kDa; Sigma‐Aldrich). Following a 4‐h fasting period with free access to water, mice received a precise dose of FITC‐dextran solution (concentration: 60 mg/100 g body weight) via orogastric gavage. Blood samples were subsequently collected after 4 h. Fasting was continued for the entire 4‐h post‐gavage period to eliminate potential interference from additional nutritional intake on intestinal permeability assessment. The collected blood was centrifuged at 4°C and 3000 ***g*** for 15 min to obtain platelet‐poor plasma. Plasma fluorescence intensity was then quantified in duplicate using a fluorescence microplate reader (BioTek Instruments, USA) with excitation and emission wavelengths set at 480 nm and 520 nm.

### 2.9 Statistical Analysis

Statistical analysis was performed using GraphPad Prism (Version 10.4.2, GraphPad Software, Boston, Massachusetts USA), with data presented as mean ± standard error of the mean. Prior to statistical testing, variance homogeneity was verified using Levene's test to ensure compliance with the assumptions of analysis of variance (ANOVA). Inter‐group differences were assessed by one‐way ANOVA followed by Tukey's post hoc test, and a *p*‐value < 0.05 was considered statistically significant.

## 3 Results

### 3.1 OA‐Rich EN Promoted Melatonin Secretion via Nutrient‐Hormone Interaction and Activated SIRT1/PGC‐1α/PPARγ Pathway After RIII

The results showed that RI significantly suppressed melatonin secretion. Specifically, melatonin concentrations in the OA‐rich EN group were 2.34‐fold higher than those in the RI group and 1.58‐fold higher than those in the RI + EN group in serum, 10.89‐fold higher than those in the RI group and 3.2‐fold higher than those in the RI + EN group in the ileum, and 3.3‐fold higher than those in the RI group and 2.0‐fold higher than those in the RI + EN group in the colon (all *p* < 0.05). These findings may suggest that OA‐rich EN, as a nutritional intervention, promoted melatonin secretion via nutritional regulation (Figure 1B–D). Then the activity of SIRT1, PGC‐1α, and PPARγ in the ileum and colon was assessed. RI led to the suppression of the SIRT1/PGC‐1α/PPARγ pathway. OA‐rich EN appeared to enhance the activity of this pathway, with relative protein expression 2.35–7.72‐fold higher than those in the RI + EN group (*p* < 0.05), exhibiting more pronounced effects than EN alone (Figure 1E–L).

### 3.2 OA‐Rich EN Alleviated Intestinal Injury and Systemic Inflammation After RIII

**FIGURE 2:**
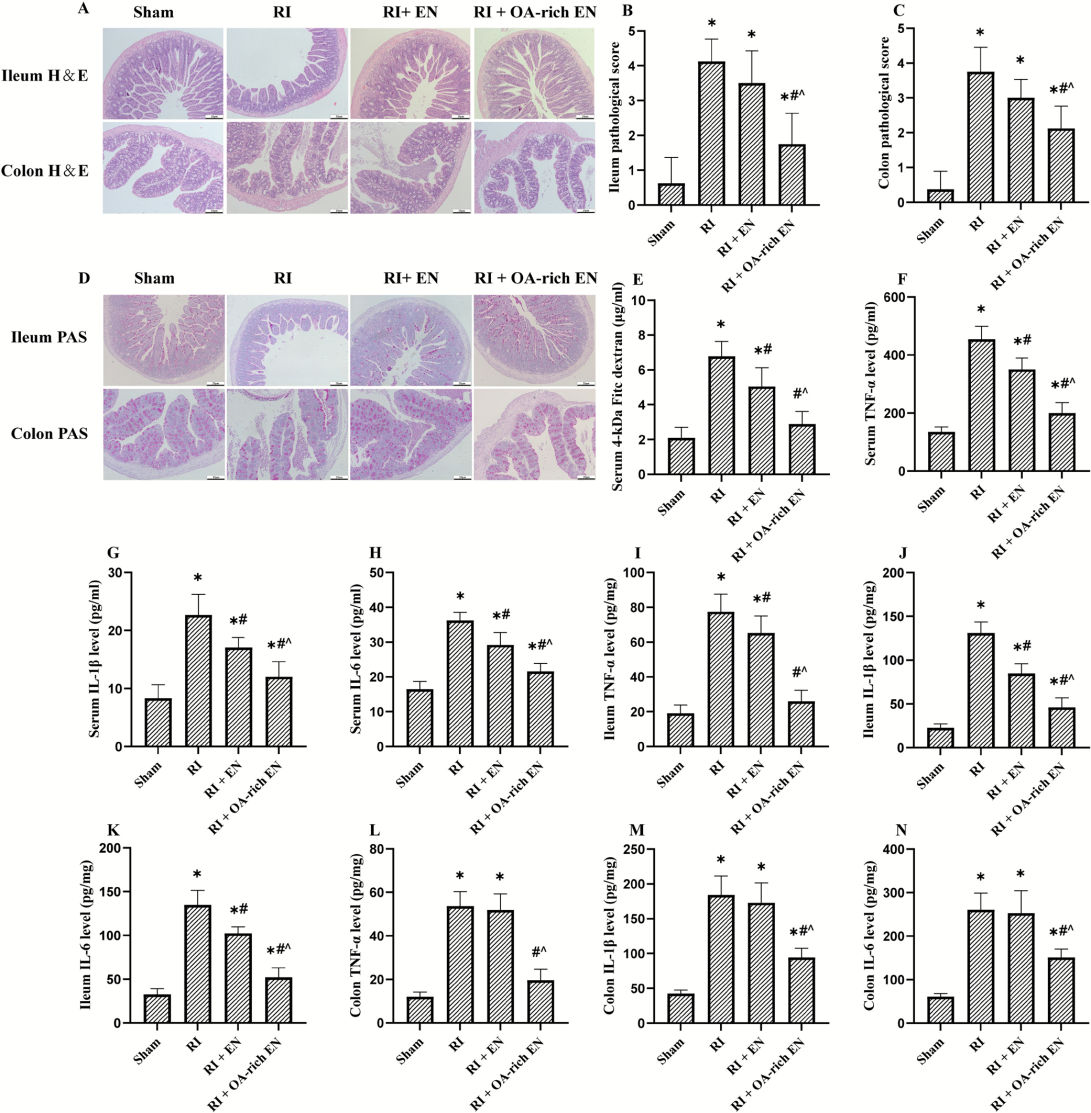
OA‐rich EN alleviated intestinal injury and systemic inflammation after RIII. H&E staining was used to evaluate general histologic changes in the ileum and colon (A). Histopathologic scoring was performed in the ileum (B) and colon (C). PAS staining was applied to assess goblet cell density and mucosal surface integrity in these regions (D). Serum level of 4‐kDa FITC‐dextran was performed to assess intestinal permeability (E). The levels of TNF‐α, IL‐1β, and IL‐6 in the serum (F‐H), ileum (I–K), and colon (L–N) were measured by ELISA. Data are presented as the mean ± standard error of the mean. *p < 0.05 versus the Sham group. #*p* < 0.05 versus the RI group. ^*p* < 0.05 versus the RI + EN group. EN, enteral nutrition; H&E, Hematoxylin–eosin; IL, interleukin; OA, octanoic acid; PAS, periodic acid‐Schiff; RI, radiation; TNF, tumor necrosis factor.

H&E revealed that RI exhibited severe architectural damage, including villous blunting, crypt distortion, and significant inflammatory cell infiltration (Figure 2A–C). Concurrently, PAS staining demonstrated a profound loss of goblet cells and attenuation of the mucin layer, suggesting a compromised mucosal barrier integrity (Figure 2D). EN intervention lowered the histopathological scores of ileum and colon, while OA supplementation further lowered the scores by an additional 29.2%–50% compared with EN alone (*p* < 0.05) and restored goblet cell density. Analysis of gut barrier function via the FITC‐dextran assay revealed that OA‐rich EN had a greater efficacy in reducing permeability, with serum fluorescence intensity 42.8% lower than in the RI + EN group, which was conducive to improving nutrient absorption (Figure 2E). Levels of TNF‐α, IL‐1β, and IL‐6 in the serum, ileum, and colon were significantly increased after RI, but these pro‐inflammatory cytokines were markedly reduced in the OA‐rich EN group, with 29.3%–60% lower values than in the RI + EN group (*p* < 0.05), which may suggest the superior efficacy of OA as a nutrient component over standard EN in mitigating radiation‐induced injury (Figure 2F–N).

### 3.3 OA‐Rich EN Alleviated Intestinal Cell Apoptosis and Intestinal Barrier Dysfunction After RIII

**FIGURE 3:**
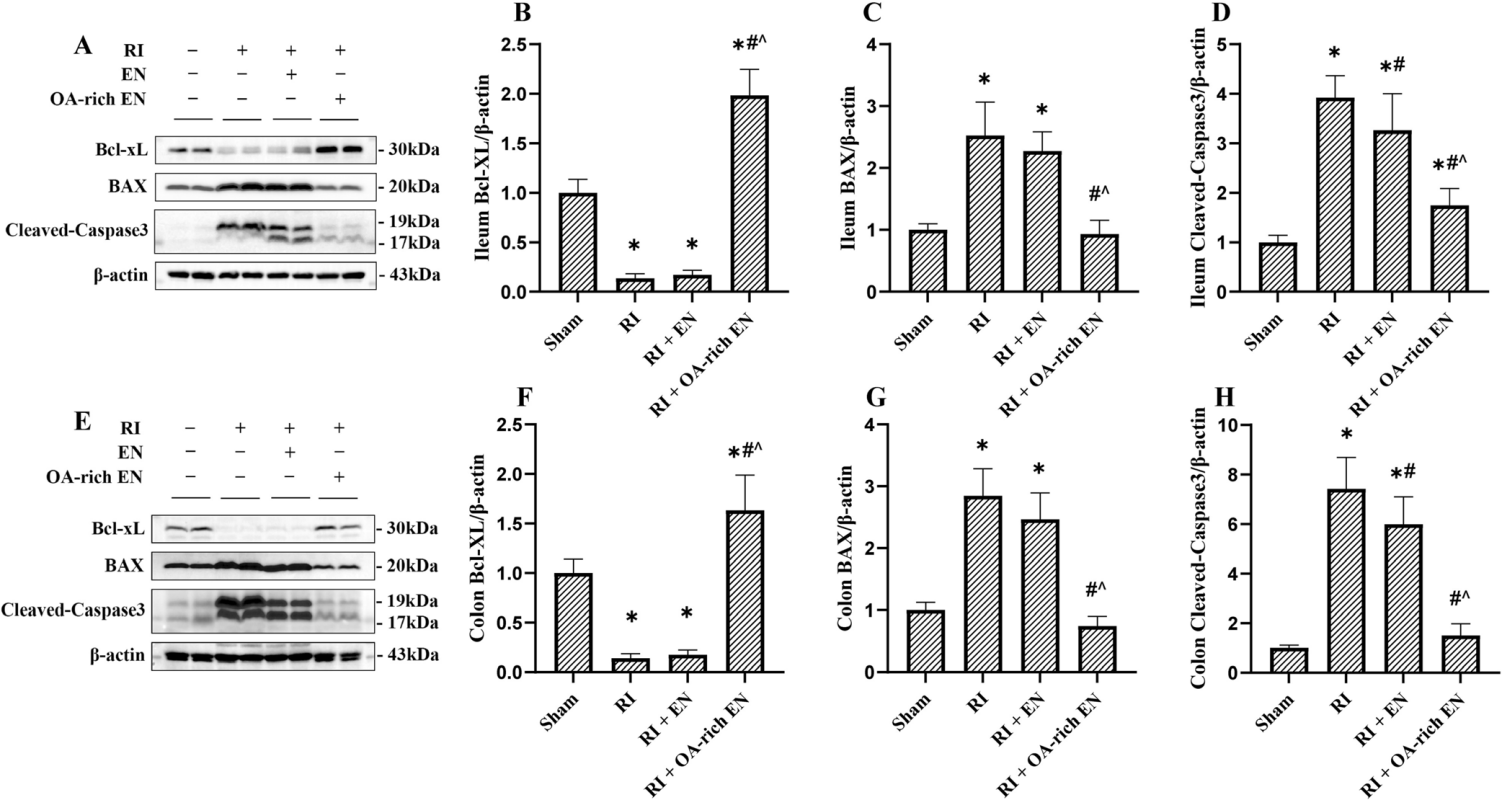
OA‐rich EN alleviated intestinal cell apoptosis after RIII. The expression levels of Bcl‐xL, BAX, and Cleaved Caspase‐3 in the ileum (A) and colon (E) were evaluated by western blotting. The relative protein expression of Bcl‐xL, BAX, and Cleaved Caspase‐3 in the ileum (B–D) and colon (F–H) was normalized to that of β‐actin. Data are presented as the mean ± standard error of the mean. *p < 0.05 versus the Sham group. #*p* < 0.05 versus the RI group. ^*p* < 0.05 versus the RI + EN group. BAX, Bcl‐2‐associated X protein; Bcl‐xL, B‐cell lymphoma‐extra large; EN, enteral nutrition; OA, octanoic acid; RI, radiation.

RI significantly downregulated the expression of the anti‐apoptotic protein Bcl‐xL, while upregulating the pro‐apoptotic proteins BAX and Cleaved Caspase‐3, indicating a marked increase in apoptosis in RIII compared with the Sham group. Both EN and OA‐rich EN interventions significantly upregulated Bcl‐xL expression and downregulated BAX and Cleaved Caspase‐3 expression. Notably, OA‐rich EN appeared to exert a significantly stronger inhibitory effect on intestinal cell apoptosis compared with EN alone, as Bcl‐xL expression was 9.35–11.61‐fold higher while BAX and Cleaved Caspase‐3 expression were reduced by 46.4%–74.9% (*p* < 0.05) in the OA‐rich EN group (Figure 3).

**FIGURE 4:**
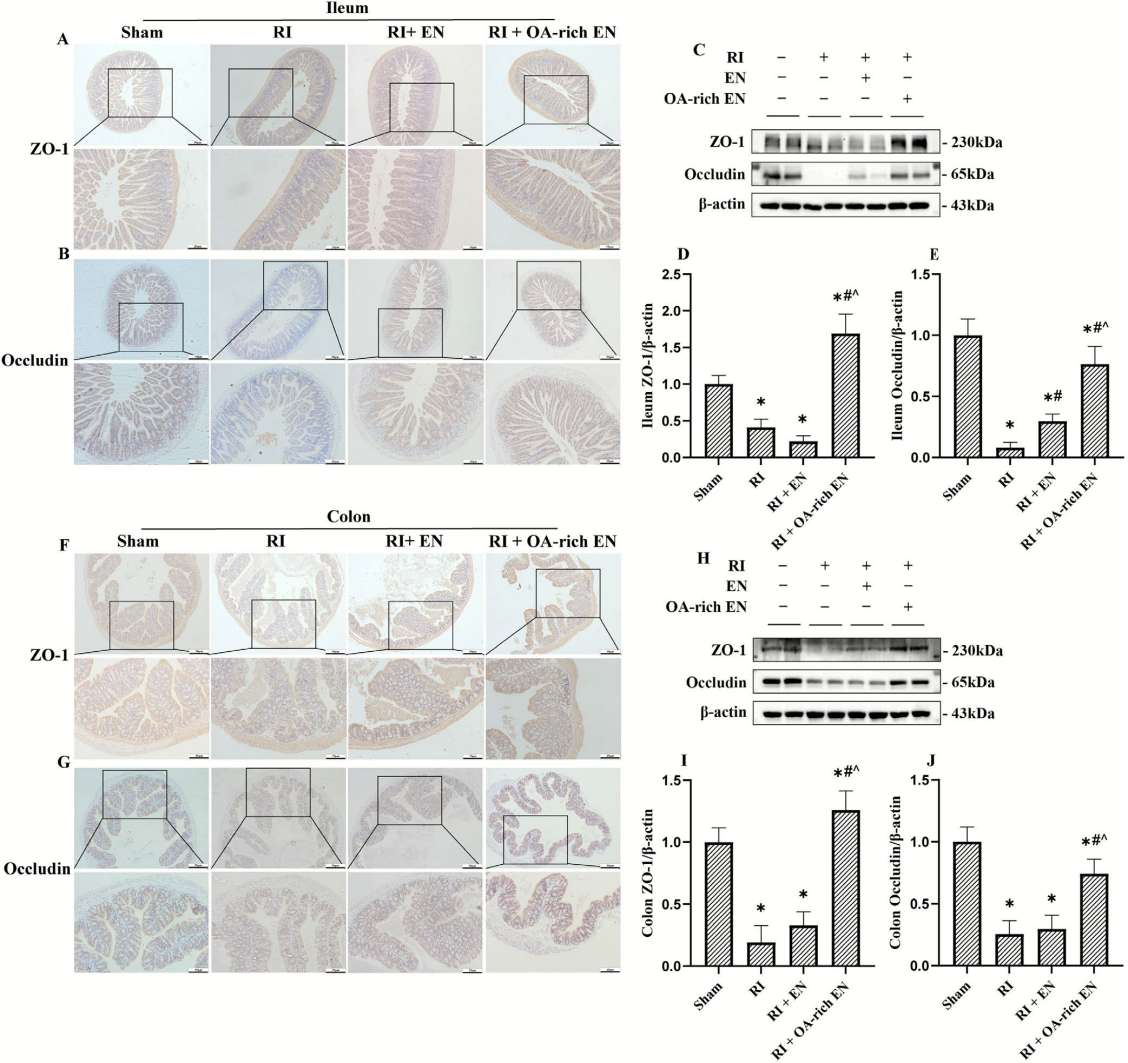
OA‐rich EN alleviated intestinal barrier dysfunction after RIII. The expression of ZO‐1 and occludin in the ileum (A–C) and colon (F–H) was evaluated by immunohistochemistry and western blotting. The relative protein expression of ZO‐1 and occludin in the ileum (D, E) and colon (I, J) was normalized to that of β‐actin. Data are presented as the mean ± standard error of the mean. *p < 0.05 versus the Sham group. #*p* < 0.05 versus the RI group. ^*p* < 0.05 versus the RI + EN group. EN, enteral nutrition; OA, octanoic acid; RI, radiation; ZO‐1, Zonula Occludens‐1.

Immunohistochemistry (Figure 4A,B,F,G) and western blot analysis (Figure 4C–E,H–J) of ZO‐1 and Occludin expression in the ileum and colon were performed to assess barrier function. RI led to the disruption of tight junction proteins ZO‐1 and Occludin, enlarged intercellular spaces, and compromised the intestinal epithelial barrier. OA‐rich EN more effectively restored the radiation‐induced decrease of tight junction proteins, with relative protein expression 2.57–7.72‐fold higher than those in the RI + EN group, which enhanced intestinal barrier integrity and promoted nutrient absorption. These findings may suggest the superior intestinal barrier protection offered by OA enrichment via nutritional regulation.

### 3.4 OA‐Rich EN Improved SIRT1/PGC‐1α/PPARγ Activity via the Melatonin‐SIRT1 Pathway

**FIGURE 5:**
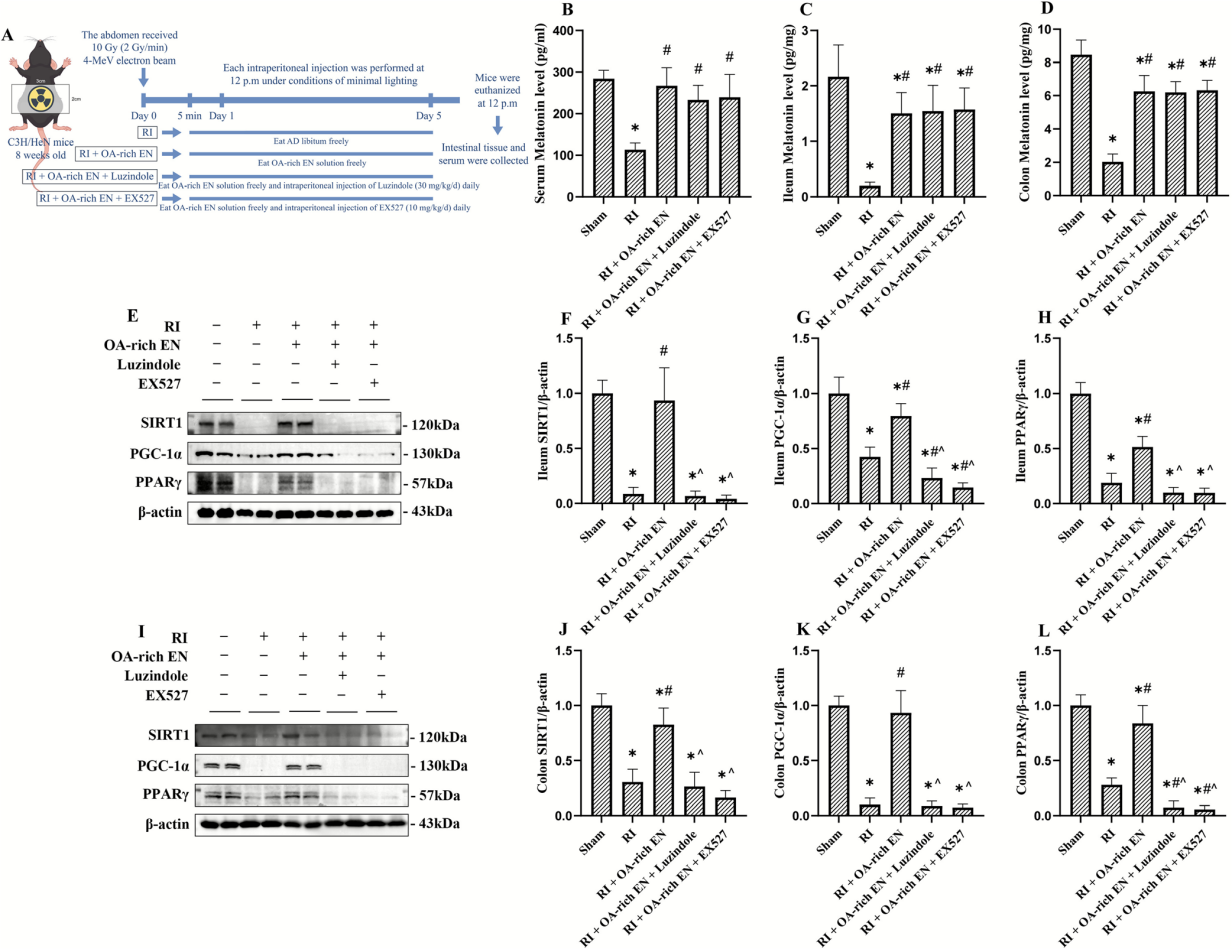
OA‐rich EN activated SIRT1/PGC‐1α/PPARγ pathway after RIII via the melatonin‐SIRT1 pathway. The grouping and treatment protocols for the mice are schematically represented (A). The levels of melatonin were measured in serum (B), ileum (C), and colon (D) by ELISA. The expression levels of SIRT1, PGC‐1α, and PPARγ in the ileum (E) and colon (I) were evaluated by western blotting. The relative protein expression of SIRT1, PGC‐1α, and PPARγ in the ileum (F–H) and colon (J–L) was normalized to that of β‐actin. Data are presented as the mean ± standard error of the mean. *p < 0.05 versus the Sham group. #*p* < 0.05 versus the RI group. ^*p* < 0.05 versus the RI + OA‐rich EN group. EN, enteral nutrition; OA, octanoic acid; PGC‐1α, Proliferator‐Activated Receptor Gamma Coactivator 1‐Alpha; PPARγ, Peroxisome Proliferator‐Activated Receptor Gamma; RI, radiation; SIRT1, Silent Information Regulator Transcript 1.

To further explore the mechanism of OA‐rich EN, a melatonin receptor inhibitor Luzindole and a SIRT1‐specific inhibitor EX527 were used. The results showed that melatonin levels were not affected by either inhibitor in the serum and intestine (Figure 5B–D). The upregulation of SIRT1/PGC‐1α/PPARγ by OA‐rich EN was counteracted by Luzindole or EX527 (Figure 5E–L).

### 3.5 OA‐Rich EN Confered Nutritional Protection Against RIII and Systemic Inflammation via the Melatonin‐SIRT1 Pathway

**FIGURE 6:**
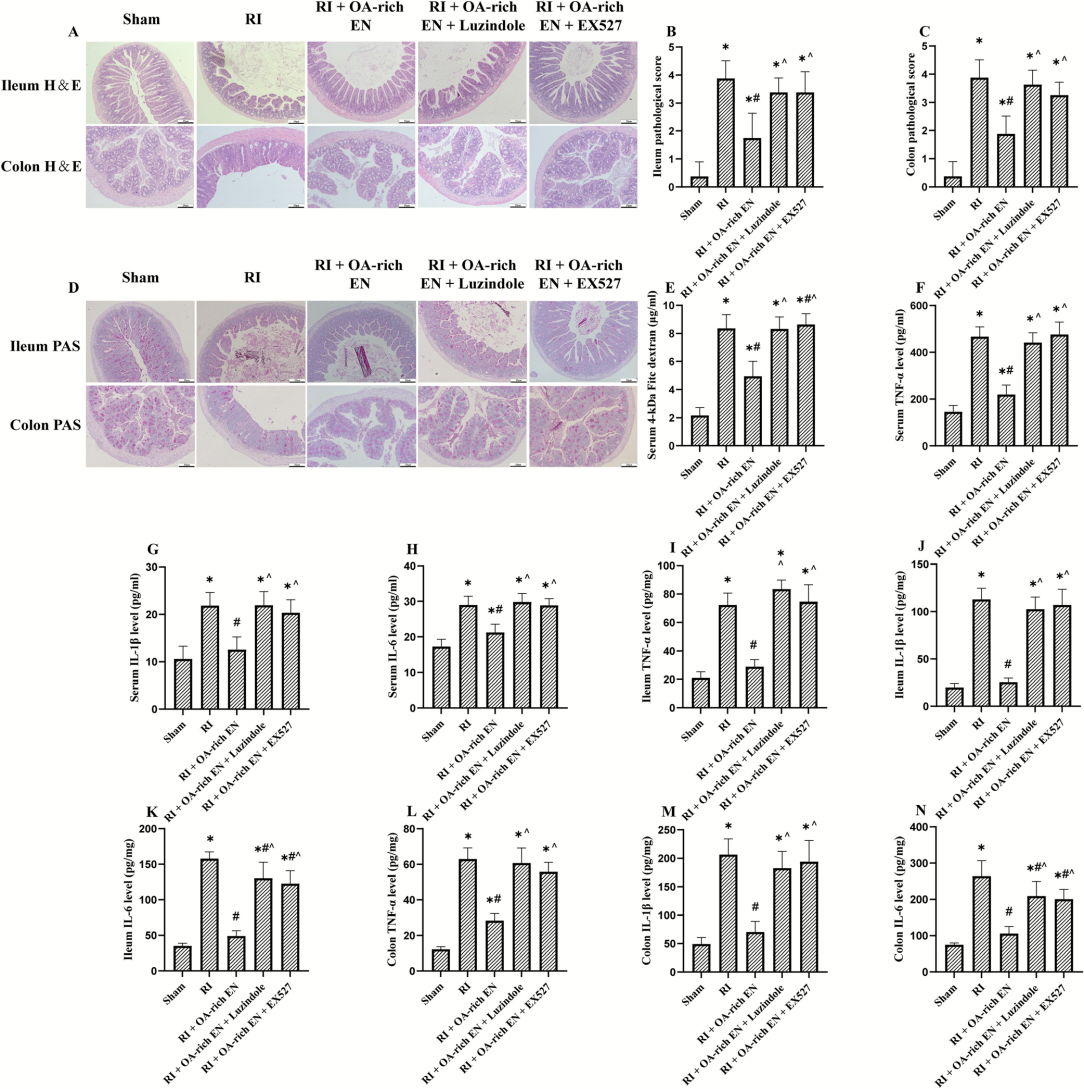
OA‐rich EN alleviated intestinal injury and systemic inflammation after RIII via the melatonin‐SIRT1 pathway. Histologic changes in the ileum and colon were evaluated with H&E staining (A). Histopathologic scoring of the ileum (B), colon (C) was performed. The goblet cell density and mucosal surface in the ileum and colon were evaluated by PAS staining (D). Serum level of 4‐kDa FITC‐dextran was performed to assess intestinal permeability (E). The levels of TNF‐α, IL‐1β, and IL‐6 in the serum (F–H), ileum (I–K), and colon (L–N) were measured by ELISA. Data are presented as the mean ± standard error of the mean. *p < 0.05 versus the Sham group. #*p* < 0.05 versus the RI group. ^*p* < 0.05 versus the RI + OA‐rich EN group. EN, enteral nutrition; H&E, Hematoxylin–eosin; IL, interleukin; OA, octanoic acid; PAS, periodic acid‐Schiff; RI, radiation; TNF, tumor necrosis factor.

Histological evaluation revealed that, compared with OA‐rich EN, the combined inhibitor treatment markedly reversed the intestinal structural improvements (Figure 6A–C). Specifically, there was recurrence of atrophy and fusion of villi, aggravated disorganization of colonic crypts, and a significant reduction in goblet cell density and mucin layer thickness (Figure 6D). Moreover, the improvement in intestinal permeability and systemic inflammation level conferred by OA‐rich EN was abolished by Luzindole or EX527 (Figure 6E–N).

### 3.6 OA‐Rich EN Inhibited Intestinal Cell Apoptosis and Intestinal Barrier Dysfunction via the Melatonin‐SIRT1 Pathway

**FIGURE 7:**
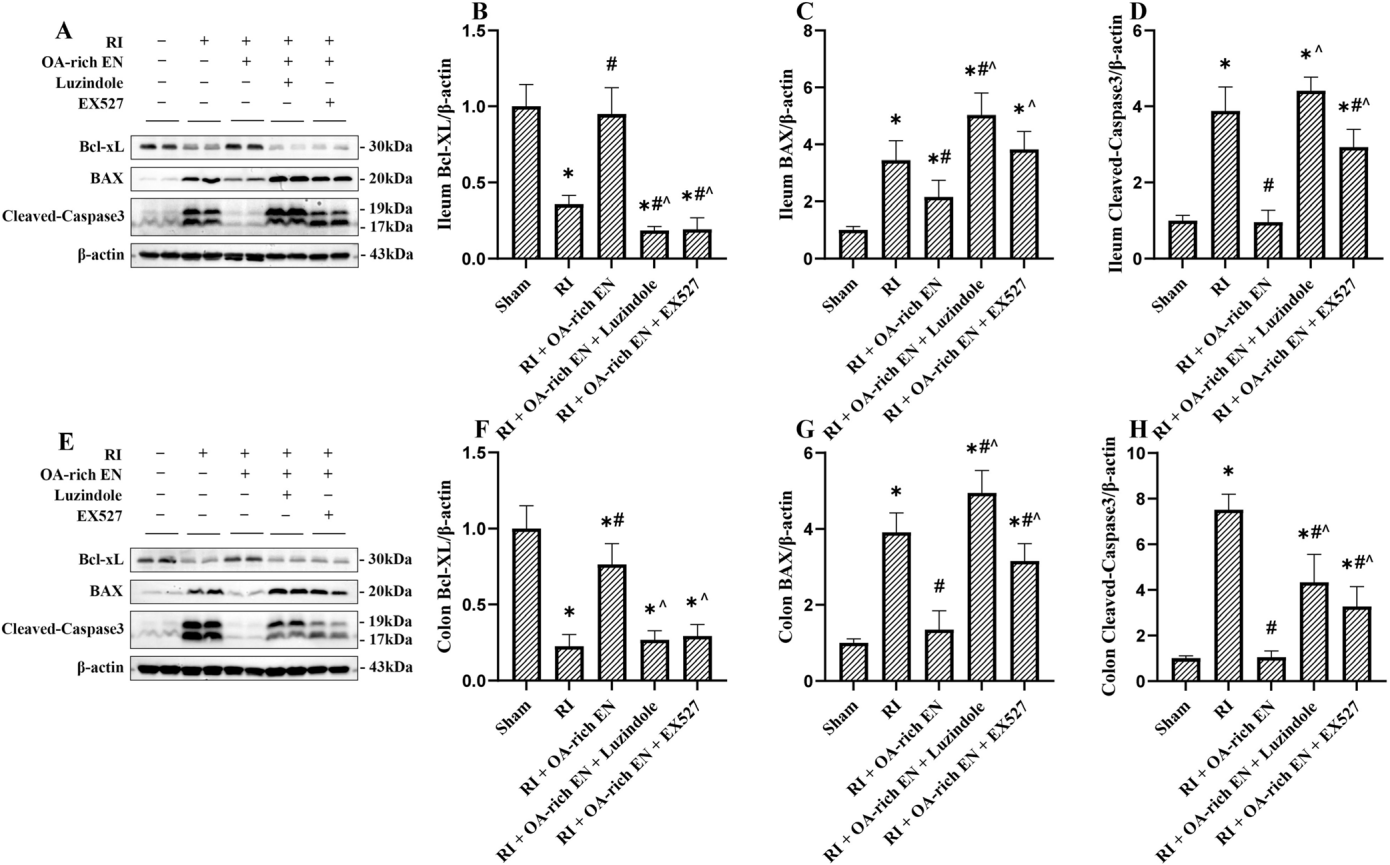
OA‐rich EN alleviated intestinal cell apoptosis after RIII via the melatonin‐SIRT1 pathway. The expression levels of Bcl‐xL, BAX, and Cleaved Caspase‐3 in the ileum (A) and colon (E) were evaluated by western blotting. The relative protein expression of Bcl‐xL, BAX, and Cleaved Caspase‐3 in the ileum (B–D) and colon (F–H) was normalized to that of β‐actin. Data are presented as the mean ± standard error of the mean. *p < 0.05 versus the Sham group. #*p* < 0.05 versus the RI group. ^*p* < 0.05 versus the RI + OA‐rich EN group. BAX, Bcl‐2‐associated X protein; Bcl‐xL, B‐cell lymphoma‐extra large; EN, enteral nutrition; OA, octanoic acid; RI, radiation.

To further substantiate this mechanism, the protein expression levels of Bcl‐xL, BAX, and Cleaved Caspase‐3 in the intestine were measured by western blot (Figure 7). The results indicated that the co‐administration of Luzindole or EX527 abolished the OA‐mediated upregulation of Bcl‐xL and downregulation of BAX and Cleaved Caspase‐3 expression, thereby preventing the alleviation of intestinal apoptosis.

**FIGURE 8:**
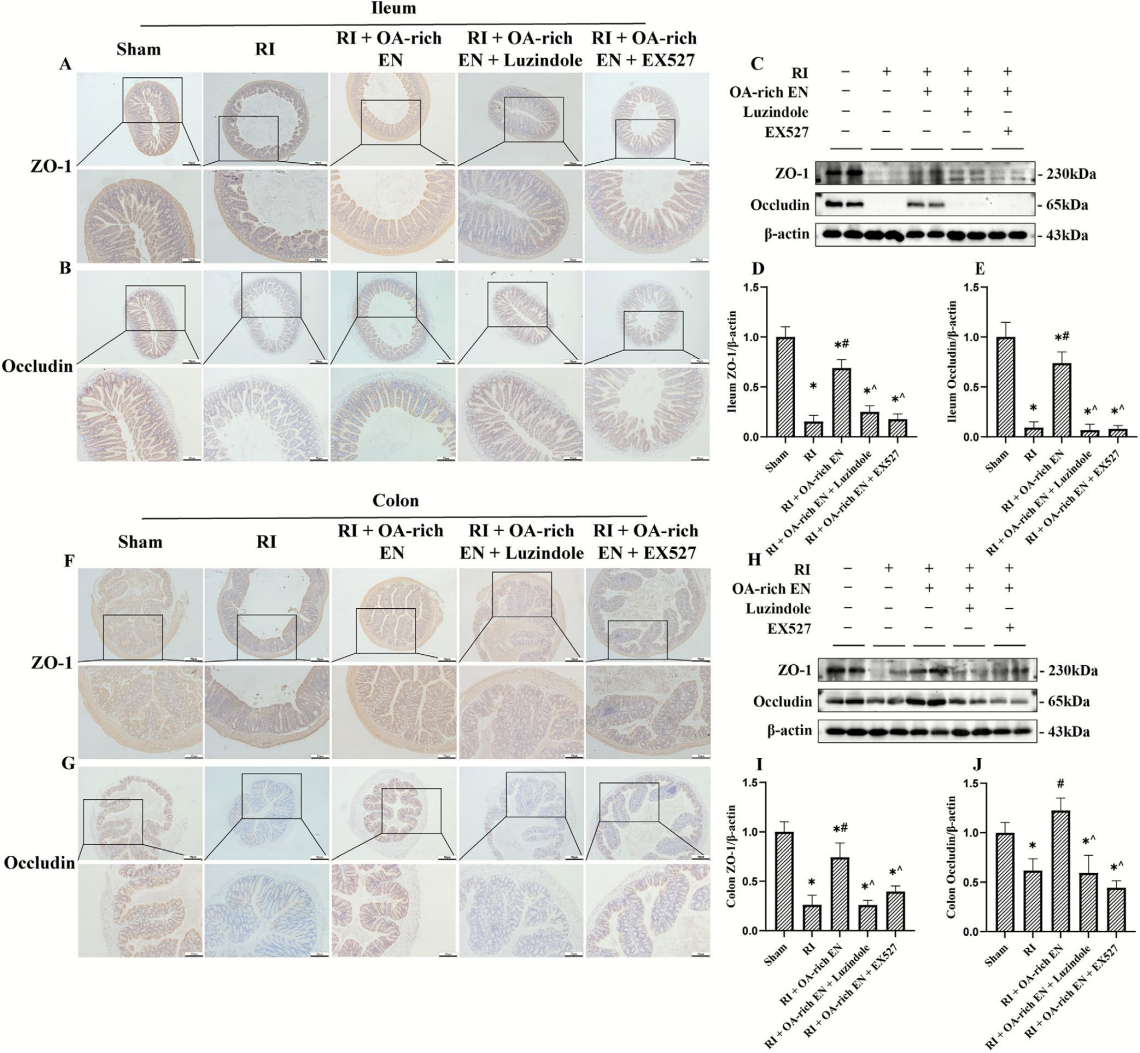
OA‐rich EN alleviated intestinal barrier dysfunction after RIII via the melatonin‐SIRT1 pathway. The expression of ZO‐1 and occludin in the ileum (A–C) and colon (F–H) was evaluated by immunohistochemistry and western blotting. The relative protein expression of ZO‐1 and occludin in the ileum (D, E) and colon (I, J) was normalized to that of β‐actin. Data are presented as the mean ± standard error of the mean. *p < 0.05 versus the Sham group. #*p* < 0.05 versus the RI group. ^*p* < 0.05 versus the RI + OA‐rich EN group. EN, enteral nutrition; OA, octanoic acid; RI, radiation; ZO‐1, Zonula Occludens‐1.

Following RI, OA‐rich EN up‐regulated the expression of the tight junction protein in ileum and colon. However, this up‐regulatory effect was abolished upon co‐administration with Luzindole or EX527 (Figure 8). This further confirms the role of the melatonin‐SIRT1 pathway in the nutritional regulatory effects of OA‐rich EN.

## 4 Discussion

RIII is a common and severe complication in patients with abdominopelvic malignancies undergoing radiotherapy, significantly impacting their quality of life and clinical prognosis (Wu et al. 2025). Nutritional assessment and interventions are considered essential for patients with abdominopelvic malignancies (Lorenzon et al. 2020; De Felice et al. 2024; Marano et al. 2023). However, an ESSO‐EYSAC global survey of surgical oncologists reveals that nutritional assessment is largely neglected in clinical practice (Lorenzon et al. 2020). And standard EN often has limited therapeutic effects—especially for RIII patients who frequently suffer from impaired nutrient absorption, energy deficiency, and exacerbated intestinal damage. This study, for the first time, suggested that OA‐rich EN conferred superior intestinal protection compared with standard EN, achieving this through nutrient‐hormone interaction: by upregulating endogenous melatonin levels, activating the SIRT1/PGC‐1α/PPARγ pathway, thereby suppressing intestinal inflammation, enhancing the intestinal mucosal barrier function, and reducing epithelial cell apoptosis.

The unique advantages of OA‐rich EN stem from the distinct nutritional metabolic properties of OA itself. Unlike long‐chain fatty acids, OA is rapidly absorbed in the small intestine without relying on bile acid emulsification, directly entering the portal circulation for rapid oxidation and energy production (Cao et al. 2025). This rapid metabolic feature allows OA to provide timely energy supply to intestinal epithelial cells damaged by radiation, supporting cell repair and proliferation. OA can also regulate hepatic metabolism by inhibiting branched‐chain alpha‐keto acid dehydrogenase kinase, promoting branched‐chain amino acid catabolism, and optimizing nutrient utilization (Kadota et al. 2015). Beyond its role as an energy source, OA acted as a specific signaling molecule with distinct regulatory effects that contribute to RIII protection independently of energy provision. This study is the first to report that OA‐rich EN promoted melatonin secretion through nutrient‐hormone interaction. Melatonin can fine‐tune the DNA damage response network, thereby reducing radiation toxicity and subsequently inhibiting cell apoptosis (Rostami et al. 2016). Notably, pre‐treatment with melatonin specifically upregulates the expression of genes involved in key DNA repair pathways, such as non‐homologous end joining (NHEJ) and homologous recombination, thereby enhancing the cellular capacity to maintain genomic stability (Valizadeh et al. 2017). This study found that melatonin levels in intestine and serum were significantly decreased in mice with RIII. The underlying mechanism may involve two aspects: firstly, radiation directly damages the intestinal mucosal epithelium and enterochromaffin cells, leading to reduced local synthesis and secretion of melatonin (Carrillo‐Vico et al. 2013; Bubenik 2002; Chen et al. 2011); secondly, the acute‐phase response to oxidative stress consumes melatonin extensively, resulting in a systemic decrease in its levels (Tang et al. 2017; Zhang and Zhang 2014). Notably, OA‐rich EN effectively elevated melatonin concentrations in intestine and serum, which linked OA's signaling‐mediated hormonal modulation to the activation of a critical radioprotective hormone pathway.

A key mechanistic target in this study was SIRT1, a member of the sirtuin family. During radiotherapy, the radiolysis of water molecules induced by radiation leads to a significant increase in reactive oxygen species levels in the extracellular environment (Kam and Banati 2013). SIRT1 extensively interacts with the endogenous antioxidant system and plays a pivotal role in regulating redox signaling and antioxidant responses. Its activation exerts protective effects on the central nervous system and RIII through its antioxidant mechanisms. Specifically, SIRT1 deacetylates PGC‐1α, facilitating the formation of a complex between FOXO3a and PGC‐1α, which subsequently upregulates the expression of various antioxidant genes (Olmos et al. 2013). On the other hand, radiation‐induced cell death is primarily attributed to irreparable DNA damage. SIRT1 can modulate the function of Ku70 via deacetylation (Jeong et al. 2007). Enhancing the interaction between SIRT1 and Ku70 helps improve the cellular capacity to repair DNA strand breaks following radiation. Furthermore, deacetylation of Ku70 reduces its dissociation from BAX, thereby stabilizing BAX in the cytoplasm and preventing its translocation to mitochondria, which in turn inhibits apoptosis (Jeong et al. 2007). Additional studies have confirmed that SIRT1 can also bind to and enhance the enzymatic activity of the neuroprotection‐associated Class I histone deacetylase, crucial for DNA repair via the NHEJ pathway (Dobbin et al. 2013). A recent study revealed that melatonin can increase the expression of SIRT1 and prevent Th17/Treg imbalance in the intestine, thereby alleviating lipopolysaccharide‐induced intestinal injury (Ma et al. 2020). Consistent with these findings, our study also demonstrated that radiation induced a decrease in SIRT1 expression in the intestine, which subsequently reduced PGC‐1α transcriptional activity and ultimately impeded the activation of PPARγ. With the increase in melatonin concentration driven by OA's hormonal modulation effect, these changes were significantly reversed: the SIRT1/PGC‐1α/PPARγ pathway was activated, the expression of pro‐inflammatory cytokines was downregulated and the activation of apoptosis was suppressed. This signaling cascade, wherein OA functions as a signaling molecule to promote melatonin secretion and subsequent activation of the SIRT1/PGC‐1α/PPARγ pathway, represents a distinct mechanism. This mechanism also aligns with our previous observations that OA‐rich EN alleviates dextran sulfate sodium salt induced inflammatory bowel disease via the PPARγ/STAT‐1/STAT‐6 pathway mediated regulation of intestinal macrophage polarization (Xue and Cao 2025). In the context of RIII, excessive activation of M1 macrophages leads to massive release of pro‐inflammatory cytokines, exacerbating intestinal mucosal damage (Zhang, Xu, et al. 2024; Zhang, Li, and Cao 2024). This study found that OA‐rich EN also activated PPARγ and significantly reduced the levels of pro‐inflammatory cytokines in the intestine and serum, suggesting that OA‐rich EN may also be able to shift the balance of intestinal macrophages from M1 to M2 in RIII. This OA's hormonal modulation effect provides a new perspective for regulating intestinal immune homeostasis in patients with RIII and enhances the anti‐inflammatory effect of the melatonin‐SIRT1 pathway activated by OA‐rich EN.

Intestinal barrier dysfunction is a key pathological feature of RIII, leading to increased permeability, reduced nutrient absorption, and systemic inflammation, all of which further worsen malnutrition and intestinal damage (Kong et al. 2023; Shao et al. 2014). This study found that OA‐rich EN significantly upregulated the expression of tight junction proteins, reduced intestinal permeability, and restored mucosal barrier integrity. The improvement of intestinal barrier function by OA‐rich EN was closely tied to its nutritional regulatory effects mediated by the melatonin‐SIRT1 pathway: on one hand, OA provided energy and essential fatty acids for the synthesis and repair of intestinal epithelial cells; on the other hand, activation of the melatonin‐SIRT1 pathway inhibited epithelial cell apoptosis and promoted mucosal regeneration. The restoration of intestinal barrier function not only prevents the translocation of intestinal bacteria and toxins but also enhances the absorption of carbohydrates, proteins, and lipids, which can improve the nutritional status of RIII patients. Furthermore, the protective effects of OA‐rich EN were markedly abolished with the administration of a melatonin receptor antagonist or a SIRT1‐specific inhibitor, confirming the central role of the melatonin‐SIRT1 pathway in mediating the protective effects of OA‐rich EN against RIII.

Currently, the pathogenesis of RIII is complex, and standardized therapeutic regimens are still lacking in clinical practice, with existing approaches primarily focusing on symptomatic and supportive care. Recent studies have explored potential interventions such as antioxidants, mesenchymal stem cells and their exosomes, and gut microbiota modulation. Furthermore, gut microbiota and their metabolites have also been demonstrated to mitigate radiation‐induced intestinal mucosal damage (Cui et al. 2017; Zheng et al. 2020). Although fecal microbiota transplantation shows therapeutic promise, it carries risks such as potential pathogen transmission and metabolite safety concerns (DeFilipp et al. 2019; Baxter et al. 2015). In contrast, OA‐rich EN, as a nutritional intervention, not only supplied energy and nutrients but also exerted active nutritional regulation through the melatonin‐SIRT1 pathway. These combined effects improve patients' nutritional status, enhance tolerance to radiotherapy, and reduce treatment‐related complications. Compared to other interventions, OA‐rich EN offers high safety, good biocompatibility and easy clinical application, making it a promising nutritional strategy for RIII. Supplementing OA based on standard EN is thus anticipated to become an effective auxiliary approach for preventing and treating radiation‐associated intestinal damage, with significant clinical promotion value.

This study has several limitations that should be considered. First, while radiation enteritis is often characterized by changes in the gut microbiota (Moraitis et al. 2023; Kumagai et al. 2018), the specific effects of OA as a dietary supplement on the microbial community were not a primary focus and thus require further exploration. Second, while inhibitor experiments provide strong evidence for the critical involvement of the melatonin‐SIRT1 axis, we acknowledge that this pathway does not exclude contributions from other nutrient‐sensing mechanisms or mitochondrial regulatory cascades, which also participate in intestinal radiation response and repair processes. Third, OA was administered at a dose established in previous literature (Zhang, Xu, et al. 2024; Zhang, Li, and Cao 2024; Tang et al. 2023). However, the absence of dose–response data represents a key limitation in translating these findings to clinical practice. In preclinical research, a single dose is often sufficient to verify biological mechanisms, but clinical application requires careful consideration of optimal dosing windows, safety margins, and interindividual variability. The metabolic capacity of OA may differ significantly between mice and humans, particularly in patients with impaired intestinal absorption. Future studies should therefore include multiple OA dosage groups to define the minimum effective dose, optimal therapeutic dose, and maximum tolerated dose. Such data would not only clarify the dose‐dependent relationship between OA supplementation and radioprotective efficacy but also provide critical guidance for setting clinical dosing schedules and safety thresholds, thereby enhancing the translational value of OA‐rich EN as a clinical intervention.

## 5 Conclusion

OA‐rich EN exerted significant protective effects against RIII via nutrient‐hormone interaction. As a key medium‐chain fatty acid with unique nutritional characteristics, OA acted as a nutrient regulator to promote melatonin secretion, which in turn activated the SIRT1/PGC‐1α/PPARγ pathway. This nutritional regulatory suppressed intestinal inflammatory responses, inhibited epithelial cell apoptosis, repaired intestinal barrier function, and improved nutrient absorption efficiency. Compared with standard EN, OA‐rich EN appeared to be associated with superior efficacy in alleviating RIII and improving intestinal health. Given the high incidence of malnutrition in patients with RIII and the limitations of current therapeutic strategies, OA‐rich EN provides a safe, effective, and clinically feasible preclinical nutritional intervention option. While these preclinical findings highlight OA‐rich EN as a promising candidate, future clinical trials are needed to validate its efficacy and optimal dosing in patients undergoing abdominal radiotherapy.

## Funding

This study was funded by the National Natural Science Foundation of China (82202369).

## Conflicts of Interest

The authors declare no conflicts of interest.

## Data Availability

The data that support the findings of this study are available on request from the corresponding author.
